# Both hypophosphatemia and hyperphosphatemia are associated with increased mortality in septic patients

**DOI:** 10.3389/fneph.2022.935288

**Published:** 2022-08-01

**Authors:** Zheng Liu, Teng Li, Yihan Du, Chenhu Li, Wei Chong

**Affiliations:** The First Affiliated Hospital of China Medical University, Shenyang, China

**Keywords:** phosphate, phosphorus, hypophosphatemia, hyperphosphatemia, normophosphatemia, sepsis

## Abstract

**Objective:**

This study was intended to explore the relationship between level of serum phosphate and prognosis in septic patients.

**Methods:**

Data were obtained from the public database, which were divided into 2 subgroups according to whether they were complicated with chronic kidney disease. Initial values of serum phosphate were extracted from patients on admission to hospital. Propensity score-matched analysis was performed. The relationship between hypophosphatemia, hyperphosphatemia and the severity of the disease in septic patients was explored separately. The lowess smoothing technique and the Kaplan-Meier method were utilized for a preliminary analysis of serum phosphate levels in relation to in-hospital mortality and 28-day survival. The initial values of serum phosphate were graded as level 1 (<1.5 mg/dL), level 2 (1.5-2.7 mg/dL), level 3 (2.7-4.5 mg/dL), level 4 (4.5-5.5 mg/dL), level 5 (5.5-6.5 mg/dL), level 6 (6.5-7.5 mg/dL) and level 7 (> 7.5 mg/dL). Multivariate logistic regression and cox regression was used to analyse the relationship between serum phosphate levels and mortality.

**Results:**

There were 4059 cases (17.4%) combined with chronic kidney disease, including 419 cases (10.3%) with hypophosphatemia and 1091 cases (26.8%) with hyperphosphatemia. There were 19224 cases (82.6%) not combined with chronic kidney disease, including 3769 cases (19.6%) hypophosphatemia and 2158 cases (11.2%) hyperphosphatemia. After propensity score-matched, in-hospital mortality, 28-day mortality, risk of septic shock was significantly higher in the 2 subgroups of hypophosphatemia patients than in normophosphatemia patients. In-hospital mortality, 28-day mortality, risk of septic shock, occurrence of renal replacement therapy, occurrence of acute renal failure, and maximum clinical score were all significantly higher in the 2 subgroups of patients with hyperphosphatemia than in patients with normophosphatemia. Multivariate logistic regression was consistent with cox regression results. In septic patients without chronic kidney disease, hypophosphatemia was an independent risk factor for death. When serum phosphate was lower, the risk of death was higher. In all septic patients, hyperphosphatemia was an independent risk factor for death. When serum phosphate was higher, the risk of death was greater.

**Conclusions:**

Both hypophosphatemia and hyperphosphatemia are associated with increased mortality in septic patients and are independent risk factors for death.

## Introduction

Sepsis has a certain morbidity and a high mortality rate (1). Although much progress has been made in the treatment of sepsis, the incidence and mortality rate of sepsis have not been significantly reduced since the surviving sepsis campaign (2). Early identification of those patients with a poor prognosis may help to provide timely and adequate treatment for sepsis (3).

Phosphate is an essential element for all living cells (4). It is also a clinically important laboratory indicator. Phosphate accounts for approximately 1% of body weight and is an essential electrolyte for the body. It has a very important physiological role. It is required for bone mineralization, the regulation of cellular energy, the synthesis of cell membranes and nucleic acids, and a variety of cellular signaling pathways (5). Of the total phosphate in the body, 85% is found in bones and teeth, 1% in extracellular fluids and 14% in cells. It is an essential component of cell membranes, nucleic acids, adenosine triphosphate (ATP) and intracellular signaling proteins (6).

Hypophosphatemia occurs in many critically ill patients, which is often indicative of severe disease (7, 8). In early studies, it has shown that hypophosphatemia is often seen in the early stages of sepsis (9, 10). Studies on relationship between serum phosphate levels and the severity of sepsis are scarce and controversial. Recent studies suggested that hyperphosphatemia rather than hypophosphatemia can reflect the prognosis of patients with sepsis (11, 12). There are also studies suggesting that severe hypophosphatemia is an independent risk factor for death in patients with sepsis (13, 14). Compared with hypophosphatemia, although few studies have focused on the relationship between hyperphosphatemia and serious disease, these studies available have concluded that hyperphosphatemia significantly increases the risk of death in septic patients (11, 12, 15). Hypophosphatemia can affect ATP synthesis, and hyperphosphatemia can promote apoptosis and inflammatory cytokines releasing oxidative stress, which may affect the prognosis of sepsis.

We hypothesized that both early hypophosphatemia and hyperphosphatemia in sepsis are factors affecting the prognosis of patients, and conducted a retrospective study of septic patients through a large database to extract initial values of serum phosphate after admission. The patients were divided into groups according to whether they had chronic kidney disease (CKD) or not, and the relationship between hypophosphatemia and hyperphosphatemia and the severity and prognosis of sepsis patients was explored.

## Materials and methods

### Database

This is a retrospective study. All data were from the Medical Information Mart for Intensive Care IV(MIMIC-IV version 1.4) database, which contains clinical information of patients in the intensive care unit (ICU) of Beth Israel Deaconess Medical Center from 2008 to 2019. The database was developed by MIT’s computational physiology laboratory and approved by the institutional review committee of MIT and Beth Israel Deaconess Medical Center. The original data were anonymous and desensitized, informed consent is not required. The data of this study was extracted by the author Liu, who completed the examination of the training plan of the cooperative organization (Record ID 45797033). Navicat Premium software (version 15.0) is used for data extraction.

### Study population and data extraction

The diagnosis of sepsis is referenced to the 2016 international guidelines sepsis 3.0 criteria (1), the data were extracted directly from the MIMIC-IV code repository (https://github.com/MIT-LCP/mimic-code/tree/main/mimic-iv/concepts/sepsis). The following patients were excluded: 1) less than 18 years old; 2) repeat hospitalized patients; 3) patients with no phosphate data; 4) the length of stay in ICU is less than 24 hours.

The following data were extracted, including demographic characteristics, types of ICUs, chronic comorbidities, laboratory indicators, scoring systems, and prognostic indicators. Demographic characteristics include age, gender, weight and smoking history. Types of ICUs include Cardiac Vascular Intensive Care Unit (CVICU), Coronary Care Unit (CCU), Medical Intensive Care Unit (MICU), Medical/Surgical Intensive Care Unit (MSICU), Neuro Intensive Care Unit (NICU), Neuro Surgical Intensive Care Unit (NSICU), Surgical Intensive Care Unit (SICU), and Trauma SICU (TSICU). Chronic comorbidities include chronic obstructive pulmonary disease, chronic heart failure, chronic kidney disease, diabetes, and malignancy. Laboratory indicators include serum phosphate values, lactate values. Scoring systems include sequential organ failure assessment score (SOFA), logistic organ dysfunction score (LODS), and simplified acute physiology score II (SAPSII). Prognostic indicators include in-hospital mortality, 28-day mortality, length of ICU stay, septic shock, mechanical ventilation, renal replacement therapy, and acute kidney injury. In this study, the lactate value is missing by 15%, we use multiple difference and compensation method to fill it, and other variables do not contain missing values.

The MIMIC-IV database defined the normophosphatemia range as 2.7-4.5 mg/dL. Therefore, 2.7-4.5 mg/dL was used as the normal range of serum phosphate in this study. In this study, a preliminary analysis was performed in three groups based on the initial serum phosphate values of the patients: the hypophosphatemia group (< 2.7 mg/dL), the normophosphatemia group (2.7-4.5 mg/dL) and the hyperphosphatemia group (>4.5 mg/dL). The normophosphatemia group was used as the reference group. In order to study the relationship between serum phosphate levels and mortality in depth, the initial serum phosphate values were classified into levels 1-7: level 1 (<1.5 mg/dL), level 2 (1.5-2.7 mg/dL), level 3 (2.7-4.5 mg/dL), level 4 (4.5-5.5 mg/dL), level 5 (5.5- 6.5 mg/dL), level 6 (6.5-7.5 mg/dL) and level 7 (>7.5 mg/dL). Initial levels of serum phosphate values were analyzed using logistic regression and cox regression according to in-hospital mortality and 28-day survival, respectively. These data were also analyzed in subgroups according to whether or not chronic kidney diseases were complicated.

### Primary and secondary outcomes

The primary outcome was in-hospital mortality. Secondary outcomes were 28-day mortality, length of ICU stay, whether septic shock occurred, whether mechanical ventilation was performed, whether renal replacement therapy was administered, and whether acute kidney injury occurred.

### Statistical analysis

The continuous variables in this study are non-normal distribution, expressed as median [interquartile range (IQR)], and the difference between groups is determined by Mann Whitney test. Categorical variables were expressed in quantity and percentage, and the comparison between groups was performed by chi square test or Fisher exact test (as appropriate).

In order to the relationship between phosphate level and prognosis, we first preliminary analyzed the unadjusted hypophosphatemia, hyperphosphatemia and reference groups, and screened out which confounding factors were statistically different among the groups. To reduce potential bias, propensity-score matching (PSM) was performed in our study by a greedy nearest neighbor matching using a caliper of 0.2 standard deviations of the logit of the estimated propensity score. Confounding factors with statistical differences were included in PSM analysis: demographic characteristics, chronic comorbidities and types of ICU admission. We used the PS graph to illustrates the matching results. Patients were matched in a 1:1 ratio, such that each patient in the hypophosphatemia group and hyperphosphatemia group was matched to one patient in the reference group.

The lowess Smoothing technique and bar graph was used to explore the crude relationship between phosphate and mortality. To determine whether phosphate levels are an independent risk factor for mortality, the multifactorial logistic regression model and cox regression model were established with the initial values of serum phosphate as design variables and the normal range (2.7-4.5 mg/dL) as the reference group. A stepwise backward elimination method with a significance level of 0.05 was used to build the final models, the variables included in the model are demographic characteristics, chronic comorbidities and types of ICU admission. Potential multicollinearity was tested using a variance inflation factor, with a value of ≥5 indicating multicollinearity. Goodness of fit was assessed for all logistic regression models. Before cox regression, the survival curve was drawn by Kaplan-Maier (K-M) method to preliminarily judge that the conditions of equal proportional risk were met. The proportional risk hypothesis test is carried out by graphical method for cox regression models. Logistic regression and cox regression were used to analyze the relationship between the dynamic change of the serum phosphate level and prognosis. Receiver operating characteristic curves (ROC) were depicted to show the diagnostic performance.

All statistical analyses were performed using the software Stata 14. All tests were two sided, and a significance level of 5% was used.

## Results

### Population and baseline characteristics

**Figure 1:**
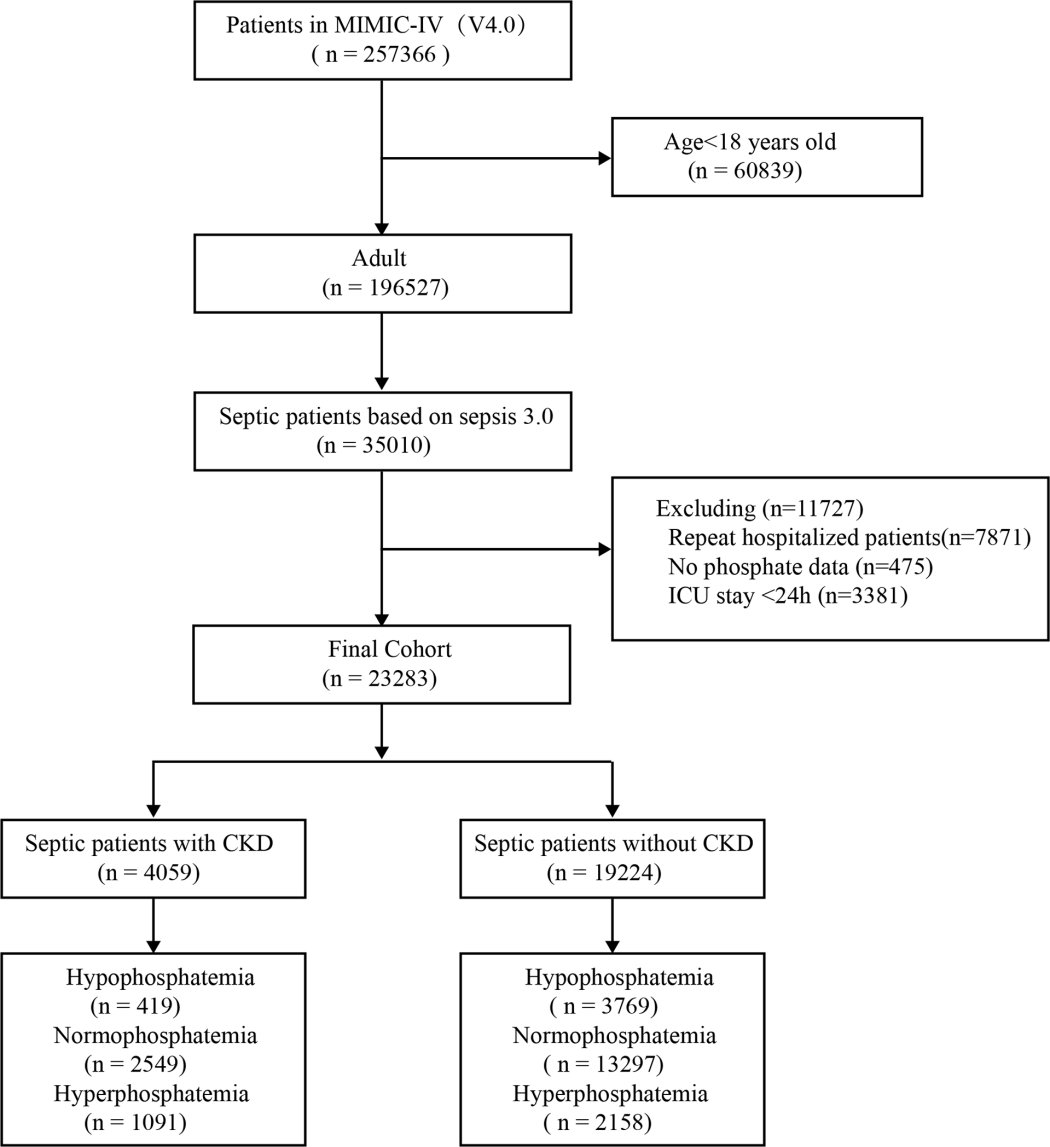
Flow chart. MIMIC-IV, Medical Information Mart for Intensive Care database; CKD, chronic kidney diseases.

A total of 23,283 septic patients were included in this study (**Figure 1**). Of these, 4059 (17.4%) cases had combined CKD and 19,224 (82.6%) cases did not have combined CKD. There were 419 (10.3%) cases of hypophosphatemia and 1091 (26.8%) cases of hyperphosphatemia in patients with CKD. There were 3769 (19.6%) hypophosphatemia and 2158 (11.2%) hyperphosphatemia in patients without CKD. With the normophosphatemia group as the reference group, the hypophosphatemia and hyperphosphatemia groups were compared with the reference group. The unadjusted results showed statistically significant differences between the hyperphosphatemia group and the normophosphatemia group for both primary and secondary outcomes (**Table 1**). There were significant differences between hypophosphatemia group and normophosphatemia group in some outcome indicators (**Table 1**).

**Table 1:** Baseline characteristics and prognosis of initial serum phosphate in septic patients with or without CKD before PSM.

	Septic patients with CKD						Septic patients without CKD			
	(n= 4,059)						(n=19,224)			
	(phosphate_0_, mg/dL)						(phosphate_0_, mg/dL)			
	<2.7	2.7-4.5		>4.5			<2.7	2.7-4.5	>4.5	
	(n= 419)	(n= 2549)		(n= 1091)		P_1_ P_2_	(n= 3769)	(n=13297)	(n=2158)	P_1_ P_2_
**Demographics**										
Age (years)	73 (63,82)		75 (65,82)		70 (59,80)	0.0736 <0.001	66 (54,76)	65 (54,76)	62 (49,74)	0.7272 <0.001
Male (n (%))	358 (85.4)		1676 (65.7)		677 (62.1)	<0.0010.978	2205 (58.5)	7220 (54.3)	1144 (53.0)	0.870 0.387
Weight, Kg (IQR)	82 (69,97)		81 (69,97)		82 (68,99)	0.7694 0.8050	78 (65,93)	81 (68,97)	86 (70,102)	<0.001<0.001
Smoking (n (%))	32 (7.6)		202 (7.9)		66 (6.1)	0.840 0.047	640 (16.9)	2341 (17.6)	312 (14.4)	0.005 <0.001
**Comorbidities (%)**										
COPD (n (%))	14 (3.3)		47 (1.8)		12 (1.2)	0.045 0.103	50 (1.3)	209 (1.6)	39 (1.8)	0.277 0.419
Malignant tumor (n (%))	51 (12.2)		199 (7.8)		66 (6.0)	0.003 0.062	386 (10.2)	1147 (8.6)	166 (7.7)	0.002 0.149
Diabetes (n (%))	206 (49.1)		1338 (52.4)		616 (56.7)	0.207 0.028	913 (24.2)	3421 (25.7)	592 (27.4)	0.061 0.094
CHF (n (%))	72 (17.1)		482 (18.9)		210 (19.2)	0.401 0.811	328 (8.7)	1272 (9.6)	251 (11.6)	0.108 0.003
**Types of ICUs**										
CVICU (n (%))	73 (17.4)		504 (19.8)		189 (17.3)	0.260 0.085	623 (16.5)	2918 (21.9)	317 (14.7)	<0.001<0.001
CCU (n (%))	46 (11.0)		406 (15.9)		205 (18.8)	0.009 0.034	229 (6.1)	1090 (8.2)	242 (11.2)	<0.001<0.001
MICU (n (%))	114 (27.2)		615 (24.1)		302 (27.9)	0.175 0.024	854 (22.7)	2619 (19.7)	602 (27.9)	<0.001 <0.001
MSICU (n (%))	96 (22.9)		456 (17.9)		181 (16.6)	0.014 0.345	870 (23.1)	2390 (17.8)	358 (16.6)	<0.001 0.119
NICU (n (%))	4 (1.0)		32 (1.2)		6 (0.5)	0.602 0.055	38 (1.0)	261 (2.0)	16 (0.7)	<0.001 <0.001
NSICU (n (%))	11 (2.6)		47 (1.8)		12 (1.1)	0.284 0.103	98 (2.6)	360 (2.7)	41 (1.9)	0.719 0.029
SICU (n (%))	53 (12.6)		309 (12.1)		120 (11.0)	0.760 0.336	593 (15.7)	1970 (14.8)	287 (13.3)	0.164 0.064
TSICU (n (%))	22 (5.2)		180 (7.1)		76 (7.0)	0.173 0.918	464 (12.3)	1689 (12.7)	295 (12.7)	0.523 0.212
**Outcome**										
Hospital Mortality (n (%))	63 (15.0)		287 (11.3)		214 (19.6)	0.026 <0.001	433 (11.5)	1186 (8.9)	477 (22.1)	<0.001 <0.001
28-Days Mortality (n (%))	59 (14.1)		271 (10.6)		215 (19.7)	0.037 <0.001	429 (11.4)	1166 (8.8)	489 (22.6)	<0.001 <0.001
ICU LOS (days) median (IQR)	3 (2,6)		3 (2,6)		3 (2,6)	0.7152 <0.001	3 (2,6)	3 (2,6)	3 (2,7)	<0.001 <0.001
SOFA score_max_ median (IQR)	4 (2,5)		3 (2,5)		4 (3,6)	0.0542 <0.001	3 (2,4)	3 (2,4)	4 (2,5)	0.2019 <0.001
SAPSII score_max_ median (IQR)	44 (35,53)		43 (35,52)		46 (37,56)	0.5286 <0.001	37 (29,46)	37 (29,46)	43 (33,54)	0.8902 <0.001
LODS score median (IQR)	6 (4,8)		6 (4,8)		6 (5,9)	0.6138 <0.001	5 (3,7)	5 (3,7)	7 (4,10)	0.7638 <0.001
Septic shock (n (%))	57 (13.6)		228 (8.9)		129 (11.8)	0.003 0.007	410 (10.9)	950 (7.1)	340 (15.8)	<0.001<0.001
MV (n (%))	211 (50.3)	1172 (45.9)		544 (49.9)		0.0960.032	2007 (53.3)	7300 (54.9)	1402 (65.0)	0.073 <0.001
RRT (n (%))	93 (22.2)	492 (19.3)		482 (44.2)		0.168 <0.001	182 (4.8)	591 (4.4)	409 (20.0)	0.285 <0.001
AKI (n (%))							737 (19.6)	2579 (19.4)	912 (42.2)	0.828 <0.001

P_1_ represents the p value of comparisons between the group with hypophosphatemia and normophosphatemia, p_2_ represents the p value of comparisons between the group with hyperphosphatemia and normophosphatemia. CKD, chronic kidney diseases; PSM, propensity-score matching; phosphate_0_, initial serum phosphate; COPD, chronic obstructive pulmonary disease; CHF, chronic heart failure; CVICU, Cardiac Vascular Intensive Care Unit; CCU, Coronary Care Unit; MICU, Medical Intensive Care Unit; MSICU, Medical/Surgical Intensive Care Unit; NICU, Neuro Intensive Care Unit; NSICU, Neuro Surgical Intensive Care Unit; SICU, Surgical Intensive Care Unit; TSICU, Trauma Surgical Intensive Care Unit; SOFA, sequential organ failure assessment; LODS, logistic organ dysfunction score; SAPSII, simplified acute physiology scoreII; MV, mechanical ventilation; RRT, renal replacement therapy; AKI, acute renal injury.

### The relationship between hypophosphatemia, hyperphosphatemia and the severity of the disease in septic patients

**Figure 2:**
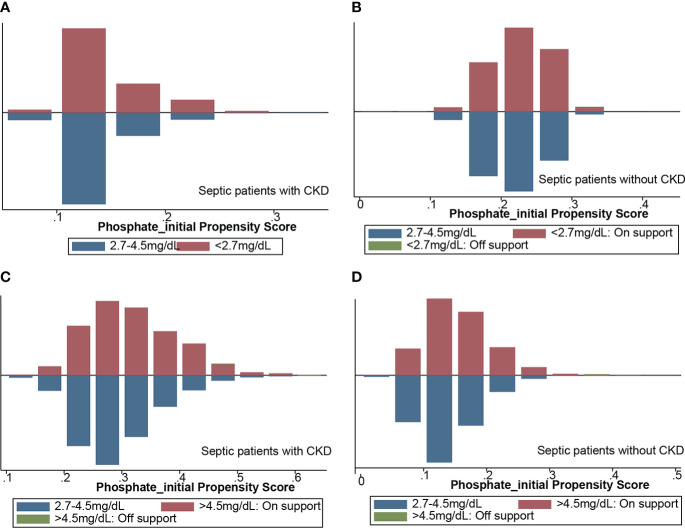
Propensity score-matched analysis of matching results. **(A)** hypophosphatemia group matched with normophosphatemia group in CKD subgroup; **(B)** hypophosphatemia group matched with normophosphatemia group in non-CKD subgroup; **(C)** hyperphosphatemia group matched with normophosphatemia in CKD subgroup; **(D)** hyperphosphatemia group matched with normophosphatemia group in non-CKD subgroup. CKD, chronic kidney diseases.

Propensity-score matching analysis:

We used the statistically different baseline characteristics in **Table 1** as a correction indicator for PSM analysis in the hypophosphatemia and normophosphatemia groups. In the CKD subgroup, 419 patients were matched to each group. In the non-CKD subgroup, 3,766 patients were matched to each group. **Figures 2A, B** showed the matching effect of PSM analysis. In the CKD subgroup, the risk of septic shock, the occurrence of mechanical ventilation, and the maximum SOFA score were significantly higher in the hypophosphatemia group than in the normophosphatemia group (**Table 2**). In the non-CKD subgroup, the hypophosphatemia group had a significantly higher risk of in-hospital mortality, 28-day mortality, risk of septic shock, and maximum SOFA score than the normophosphatemia group (**Table 2**). We used the baseline characteristics that were statistically different in **Table 1** as corrected indicators for PSM analysis in the hyperphosphatemia and normophosphatemia groups. In the CKD subgroup, 1,087 patients were matched to each group. In the non-CKD subgroup, 2155 patients were matched to each group. **Figures 2C, D** showed the matching effect of PSM analysis. In the CKD subgroup, there was no statistical difference in the occurrence of mechanical ventilation (p=0.071). In both two subgroups, in-hospital mortality, 28-day mortality, risk of septic shock, occurrence of renal replacement therapy, and maximum clinical score were significantly higher in the hyperphosphatemia group than in the normophosphatemia group (**Table 3**). The incidence of acute kidney injury (AKI) was significantly higher in the hyperphosphatemia group than in the normophosphatemia group in the non-CKD subgroup (**Table 3**).

**Table 2:** Comparison of prognosis between hypophosphatemia and normophosphatemia in septic patients with or without CKD after PSM.

	Septic patients with CKD			Septic patients without CKD		
	(phosphate_0_, mg/dL)			(phosphate_0_, mg/dL)		
	2.7-4.5	<2.7		2.7-4.5	<2.7	
	(n=419)	(n=419)	P	(n=3766)	(n=3766)	P
**Outcome**						
Hospital Mortality (n (%))	51 (12.2)	63 (15.0)	0.227	340 (9.0)	432 (11.5)	<0.001
28-Days Mortality (n (%))	45 (10.7)	59 (14.0)	0.142	331 (8.8)	428 (11.4)	<0.001
ICU LOS (days) median (IQR)	3 (2,6)	3 (2,6)	0.1247	3 (2,6)	3 (2,6)	0.5343
SOFA score_max_ median (IQR)	3 (2,5)	4 (2,5)	0.0076	3 (2,4)	4 (2,5)	0.0120
SAPSII score_max_ median (IQR)	43 (35,51)	44 (35,53)	0.3871	36 (28,47)	37 (29,46)	0.6981
LODS score median (IQR)	6 (4,8)	6 (4,8)	0.1149	5 (3,7)	5 (3,7)	0.5080
Septic shock (n (%))	35 (8.4)	57 (13.6)	0.015	286 (8.0)	410 (10.9)	<0.001
MV (n (%))	182 (43.7)	211 (50.4)	0.045	1992 (52.9)	2006 (53.2)	0.747
RRT (n (%))	74 (17.7)	93 (22.2)	0.100	149 (4.0)	183 (4.9)	0.056
AKI (n (%))				748 (19.9)	737 (19.6)	0.750

CKD, chronic kidney diseases; PSM, propensity-score matching; phosphate_0_, initial serum phosphate; SOFA, sequential organ failure assessment; LODS, logistic organ dysfunction score; SAPSII, simplified acute physiology scoreII; MV, mechanical ventilation; RRT, renal replacement therapy; AKI, acute renal injury.

**Table 3:** Comparison of prognosis between hyperphosphatemia and normophosphatemia in septic patients with or without CKD after PSM. .

	Septic patients with CKD			Septic patients without CKD		
	(phosphate_0_, mg/dL)			(phosphate_0_, mg/dL)		
	2.7-4.5	>4.5		2.7-4.5	>4.5	
	(n=1087)	(n=1087)	P	(n=2155)	(n=2155)	P
**Outcome**						
Hospital Mortality (n (%))	107 (9.8)	213 (19.6)	<0.001	207 (9.6)	477 (22.1)	<0.001
28-Days Mortality (n (%))	105 (9.7)	214 (19.7)	<0.001	198 (9.2)	489 (22.7)	<0.001
ICU LOS (days) median (IQR)	3 (2,6)	3 (2,6)	0.0109	3 (2,6)	3 (2,7)	0.0023
SOFA score_max_ median (IQR)	3 (2,5)	4 (3,6)	<0.001	3 (2,4)	4 (2,5)	<0.001
SAPSII score_max_ median (IQR)	41 (34,50)	46 (37,56)	<0.001	36 (28,46)	43 (33,54)	<0.001
LODS score median (IQR)	6 (4,8)	6 (5,9)	<0.001	5 (3,8)	7 (4,10)	<0.001
Septic shock (n (%))	88 (8.1)	129 (11.9)	0.003	187 (8.7)	430 (20.0)	<0.001
MV (n (%))	500 (45.0)	542 (49.9)	0.071	1221 (56.7)	1400 (65.0)	<0.001
RRT (n (%))	235 (21.6)	478 (44.0)	<0.001	128 (5.9)	409 (19.0)	<0.001
AKI (n (%))				482 (22.4)	910 (42.2)	<0.001

CKD, chronic kidney diseases; PSM, propensity-score matching; phosphate_0_, initial serum phosphate; SOFA, sequential organ failure assessment; LODS, logistic organ dysfunction score; SAPSII, simplified acute physiology scoreII; MV, mechanical ventilation; RRT, renal replacement therapy; AKI, acute renal injury.

### The relationship between serum phosphate values and mortality

#### Lowess smoothing and bar graph

**Figure 3:**
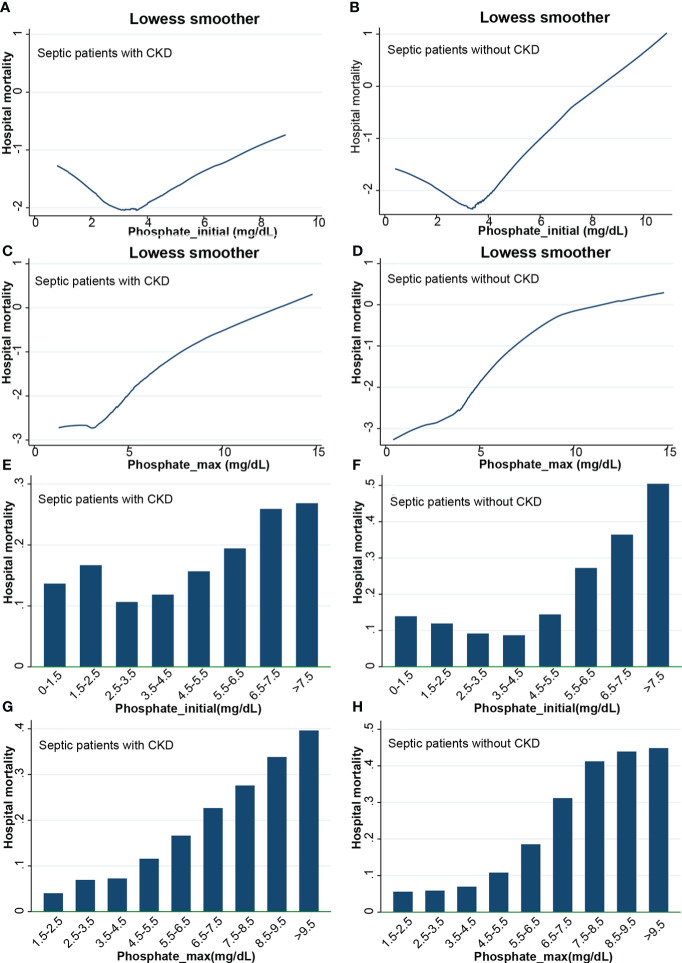
Lowess curves and bar graphs. **(A)** Lowess curve of relationship between initial phosphate values and in-hospital mortality in CKD subgroup; **(B)** Lowess curve of relationship between initial phosphate values and in-hospital mortality in non-CKD subgroup; **(C)** Lowess curve of relationship between maximum phosphate values and in-hospital mortality in CKD subgroup; **(D)** Lowess curve of relationship between maximum phosphate values and in-hospital mortality in non-CKD subgroup; **(E)** Bar graph of relationship between initial phosphate values and in-hospital mortality in CKD subgroup; **(F)** Bar graph of relationship between initial phosphate values and in-hospital mortality in non-CKD subgroup; **(G)** Bar graph of relationship between maximum phosphate values and in-hospital mortality in CKD subgroup; **(H)** Bar graph of relationship between maximum phosphate values and in-hospital mortality in non-CKD subgroup. CKD, chronic kidney diseases.

The lowess smoothing technique was used to roughly assess the relationship between initial serum phosphate values and in-hospital mortality (**Figures 3A, B**). In two subgroups, in-hospital mortality showed a U-shaped relationship with serum phosphate levels. As serum phosphate levels increased, in-hospital mortality decreased and then increased. The bar graph showed that in-hospital mortality was lowest in the CKD subgroup with serum phosphate levels of 2.5-3.5 mg/dL (**Figure 3E**). In the non-CKD subgroup, in-hospital mortality was lowest at a serum phosphate of 3.5-4.5 mg/dL (**Figure 3F**). The lowess smoothing technique was used to roughly assess the relationship between the maximum value of serum phosphate and in-hospital mortality (**Figures 3C, D**). In-hospital mortality was linearly related to serum phosphate levels in the two subgroups. In-hospital mortality increased with increasing levels of serum phosphate. The bar graph is consistent with the trend of the lowess curve (**Figures 3G, H**).

#### Logistic regression analysis

**Figure 4:**
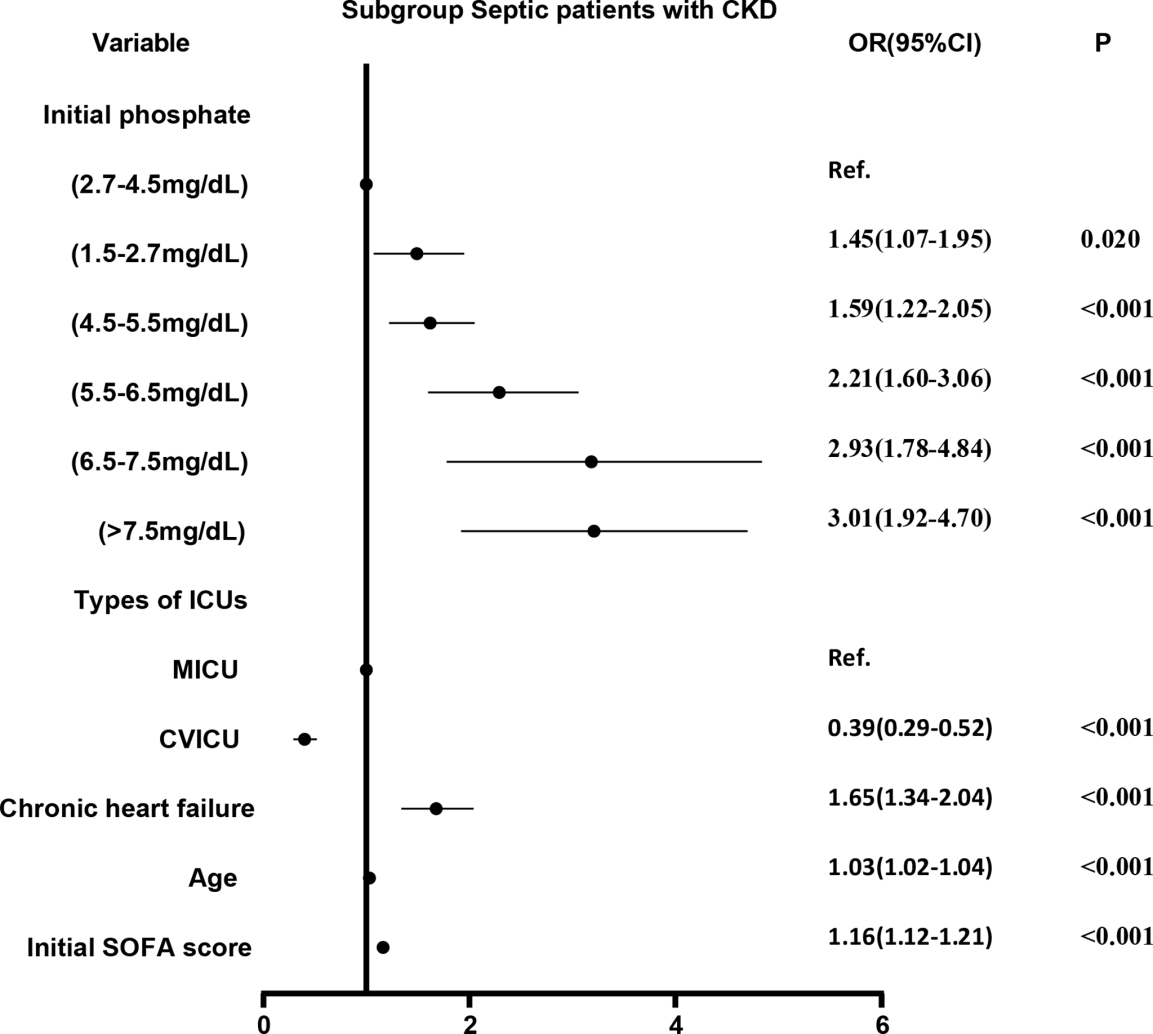
The adjusted odds ratio using initial phosphate values as the design variable in CKD subgroup. The stepwise backward method corrects the confounding factors and removes the variables in turn: phosphate_initial<1.5 mg/dL (p = 0.8286), smoking (p = 0.6233), NICU (p = 0.5527), COPD (p = 0.4875), malignant tumor (p = 0.3060), gender (p = 0.2421), MSICU (p = 0.1956), SICU (p = 0.2764), CCU (p = 0.1414), TSICU (p = 0.1603), NSICU (p = 0.0940), and weight (p = 0.0610), the mean VIF was 2.98. CKD, chronic kidney diseases; COPD, chronic obstructive pulmonary disease; CVICU, Cardiac Vascular Intensive Care Unit; CCU, Coronary Care Unit; MICU, Medical Intensive Care Unit; MSICU, Medical/Surgical Intensive Care Unit; NICU, Neuro Intensive Care Unit; NSICU, Neuro Surgical Intensive Care Unit; SICU, Surgical Intensive Care Unit; TSICU, Trauma Surgical Intensive Care Unit; SOFA, sequential organ failure assessment.

**Figure 5:**
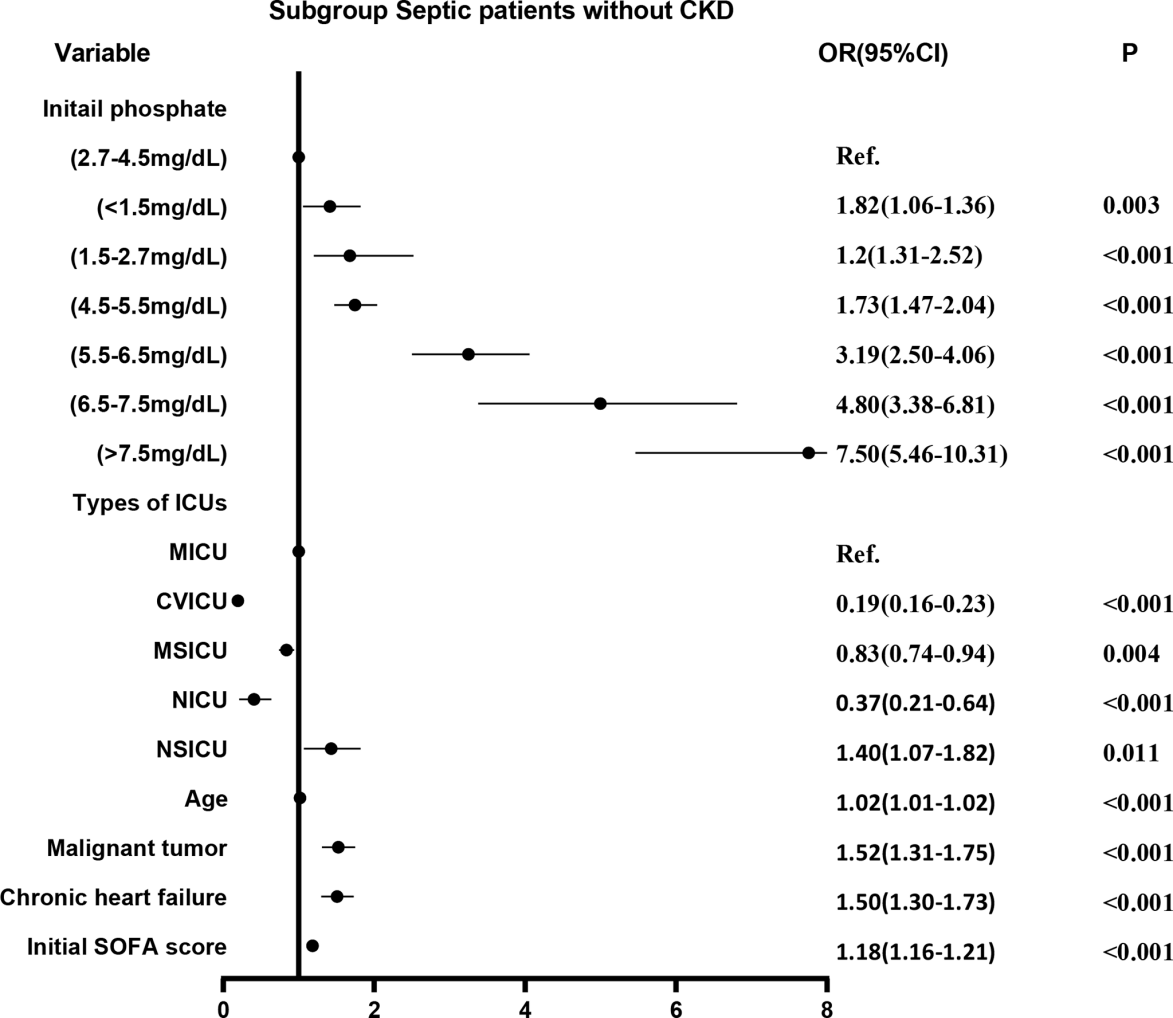
The adjusted odds ratio using initial phosphate values as the design variable in non-CKD subgroup. The stepwise backward method corrects the confounding factors and removes the variables in turn: CCU (p = 0.6239), gender (p = 0.4071), smoking (p = 0.3037), weight (p = 0.2000), COPD (p = 0.1518), SICU (p = 0.1266), and TSICU (p = 0.0644), the mean VIF was 1.56. CKD, chronic kidney diseases; COPD, chronic obstructive pulmonary disease; CVICU, Cardiac Vascular Intensive Care Unit; CCU, Coronary Care Unit; MICU, Medical Intensive Care Unit; MSICU, Medical/Surgical Intensive Care Unit; NICU, Neuro Intensive Care Unit; NSICU, Neuro Surgical Intensive Care Unit; SICU, Surgical Intensive Care Unit; TSICU, Trauma Surgical Intensive Care Unit; SOFA, sequential organ failure assessment.

We divided the initial values of serum phosphate from low to high into 7 levels (as described above). Level 3 (2.7-4.5 mg/dL) was used as the reference group and the other levels were used as dummy variables. Logistic regression analysis was performed on the 7 levels, with stepwise backward correction for covariates. In the subgroup of CKD, all but level 1 (<1.5 mg/dL) were independent risk factors for in-hospital mortality. The OR was greater as the level increased (**Figure 4**). In the non-CKD subgroup, all levels were independent risk factors for in-hospital mortality. The lower the serum phosphate at hypophosphatemia, the greater the OR. The higher the serum phosphate at hyperphosphatemia, the greater the OR (**Figure 5**).

#### Cox regression analysis

**Figure 6:**
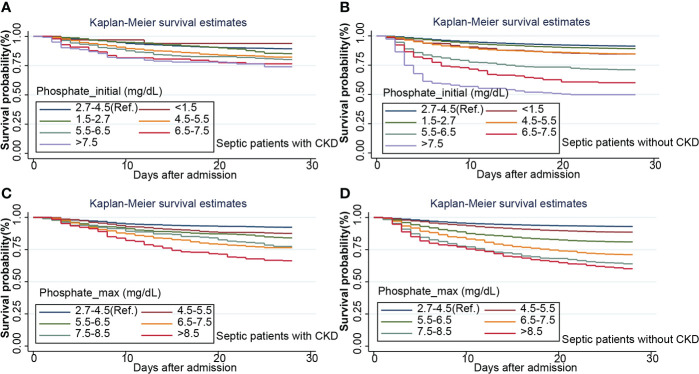
Kaplan-Maier survival curve describes the relationship between initial phosphate value **(A, B)** or maximum phosphate value **(C, D)** in two subgroups and 28-day survival rate in septic patients. CKD, chronic kidney diseases.

**Figure 7:**
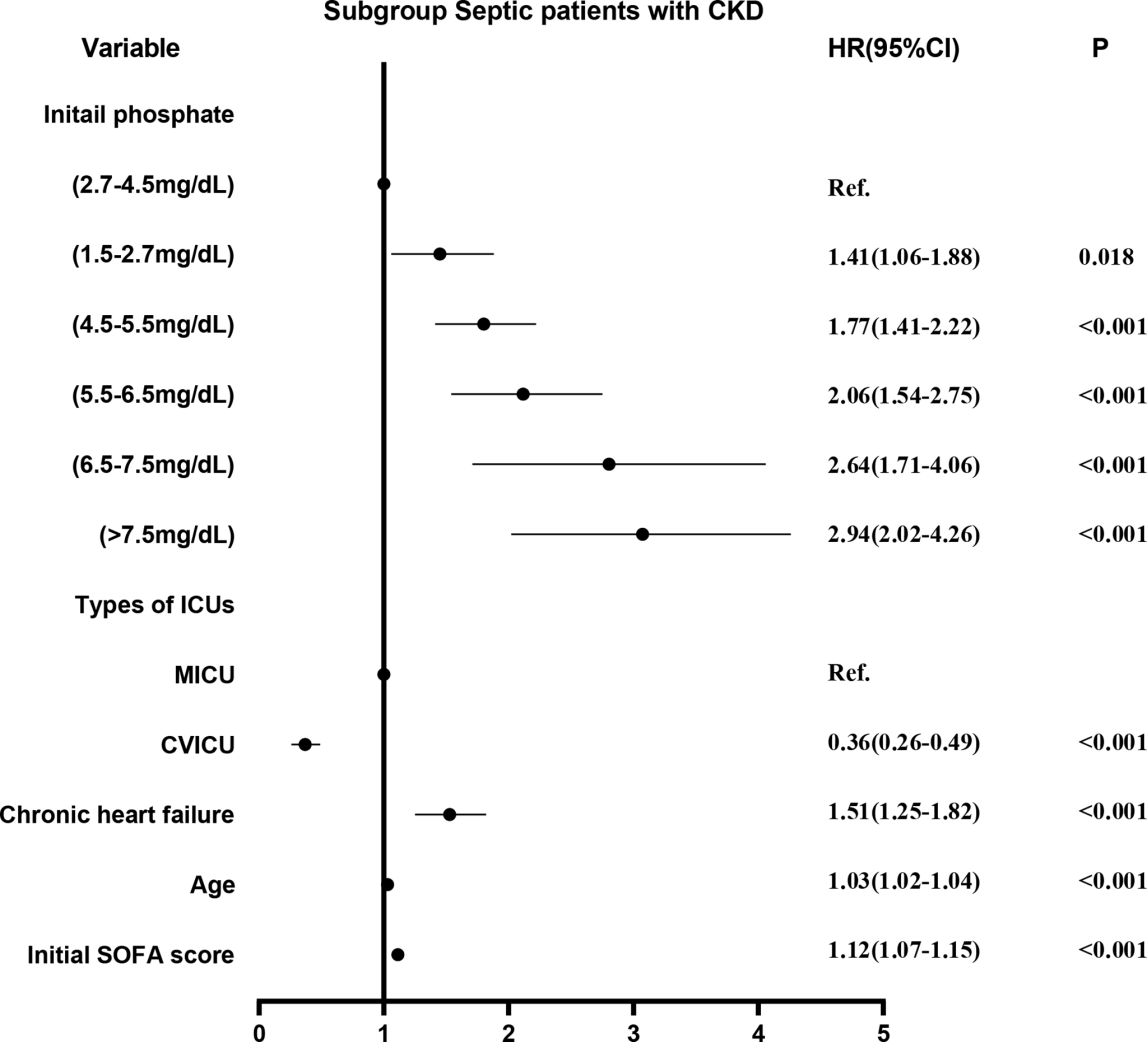
The adjusted hazard ratio using initial phosphate values as the design variable in CKD subgroup. The stepwise backward method corrects the confounding factors and removes the variables in turn: smoking (p = 0.8411), malignant tumor (p = 0.7799), weight (p = 0.5289), NICU (p = 0.4821), COPD (p = 0.4638), phosphate_initial<1.5 mg/dL (p = 0.4210), SICU (p = 0.4096), MSICU (p = 0.4656), gender (p = 0.3673), CCU (p = 0.3195), TSICU (p = 0.2907), and NSICU (p = 0.2787). CKD, chronic kidney diseases; COPD, chronic obstructive pulmonary disease; CVICU, Cardiac Vascular Intensive Care Unit; CCU, Coronary Care Unit; MICU, Medical Intensive Care Unit; MSICU, Medical/Surgical Intensive Care Unit; NICU, Neuro Intensive Care Unit; NSICU, Neuro Surgical Intensive Care Unit; SICU, Surgical Intensive Care Unit; TSICU, Trauma Surgical Intensive Care Unit; SOFA, sequential organ failure assessment.

**Figure 8:**
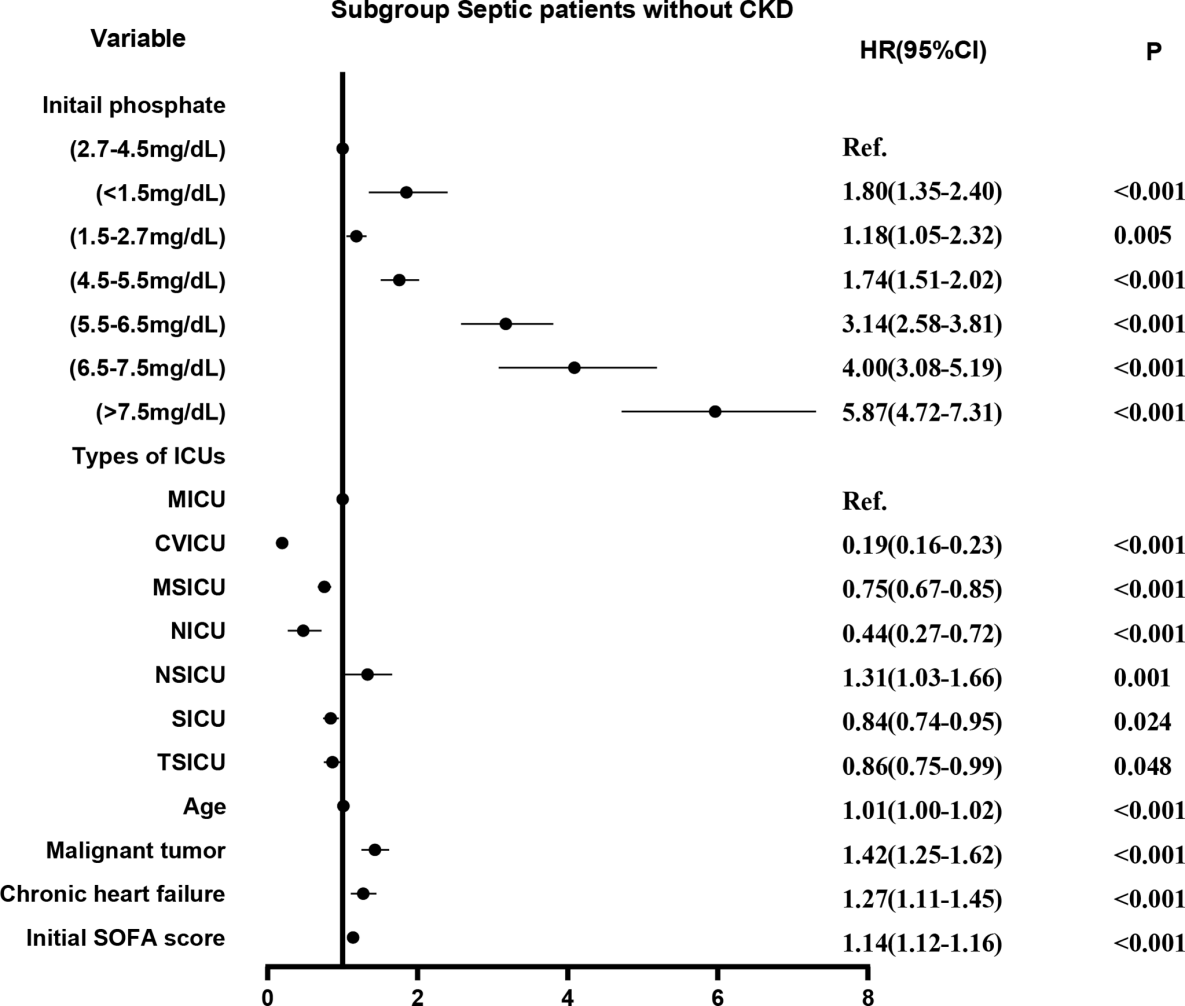
The adjusted hazard ratio using initial phosphate values as the design variable in non-CKD subgroup. The stepwise backward method corrected for confounding factors and removed the variables in turn: CCU (p = 0.8841), gender (p = 0.8806), smoking (p = 0.4945), COPD (p = 0.2086), and weight (p = 0.1044). CKD, chronic kidney diseases; COPD, chronic obstructive pulmonary disease; CVICU, Cardiac Vascular Intensive Care Unit; CCU, Coronary Care Unit; MICU, Medical Intensive Care Unit; MSICU, Medical/Surgical Intensive Care Unit; NICU, Neuro Intensive Care Unit; NSICU, Neuro Surgical Intensive Care Unit; SICU, Surgical Intensive Care Unit; TSICU, Trauma Surgical Intensive Care Unit; SOFA, sequential organ failure assessment.

Survival curves were first plotted using the K-M method, with the aim of making a preliminary determination of the relationship between the initial and maximum values of serum phosphate and survival as well as whether the equiproportional risk condition required for cox regression was met (**Figure 6**). Cox regression analysis was performed on the seven groups of initial serum phosphate values, with level 3 (2.7-4.5 mg/dL) as the reference group and the other levels as dummy variables, corrected for covariates by the stepwise backward method. In the subgroup with CKD, all but level 1 (<1.5 mg/dL) were independent risk factors for 28-day mortality. When the level was higher, the HR value was higher (**Figure 7**). In the non-CKD subgroup, all levels were independent risk factors for 28-day mortality. In hyperphosphatemia, the HR was greater when serum phosphate was higher. The higher the serum phosphate at hyperphosphatemia, the greater the HR (**Figure 8**).

### Relationship between the dynamic change of the serum phosphate level and prognosis

Compared with the normophosphatemia during hospitalization, the change of serum phosphate will affect the prognosis (**Table 4**).

**Table 4:** Adjusted ORs and HRs using the dynamic change of serum phosphate levels as the design variable.

Model1			Model2		
Variable	OR (95%CI)	P	Variable	HR (95%CI)	P
Norm to norm	Ref.		Norm to norm	Ref.	
Norm to low	1.45 (1.24-1.69)	<0.001	Norm to low	1.48 (1.28-1.71)	<0.001
Norm to high	4.61 (4.00-5.30)	<0.001	Norm to high	3.68 (3.25-4.17)	<0.001
Low to norm	1.25 (1.07-1.46)	0.004	Low to norm	1.23 (1.06-1.43)	0.005
Low to low	1.86 (1.55-2.23)	<0.001	Low to low	1.88 (1.60-2.22)	<0.001
Low to high	5.60 (4.31-7.27)	<0.001	Low to high	3.64 (2.89-4.57)	<0.001
High to norm	1.65 (1.40-1.95)	<0.001	High to norm	1.72 (1.48-2.01)	<0.001
High to low	3.45 (2.56-4.65)	<0.001	High to low	3.19 (2.49-4.11)	<0.001
High to high	6.71 (5.78-7.80)	<0.001	High to high	5.94 (5.25-6.72)	<0.001

Model1: logistic regression analysis, the mean VIF was 1.55.

Model2: cox regression analysis.

Model1 and model2 used stepwise backward method was to correct the confounding factors including demographic characteristics, chronic comorbidities and types of ICU admission.

norm, normophosphatemia; low, hypophosphatemia; high, hyperphosphatemia.

### Predictive effect of serum phosphate on death of septic patients

**Figure 9:**
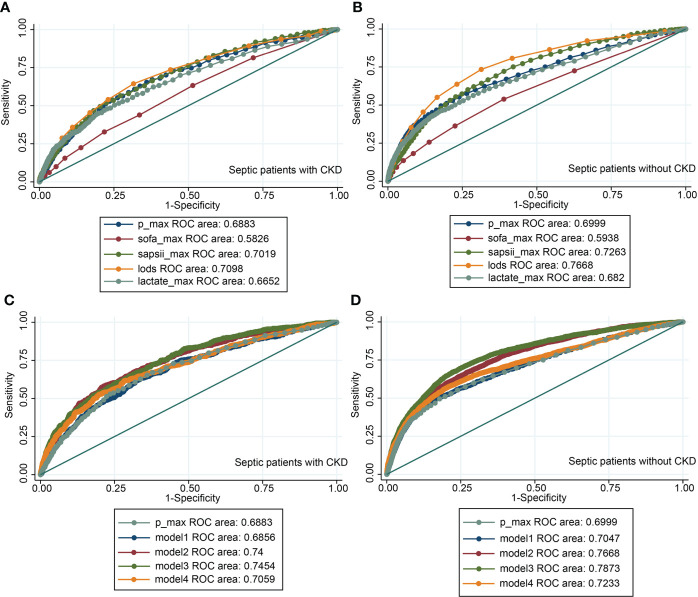
The receiver operating characteristic curves. **(A)** ROC of the maximum phosphate value, the maximum clinical score, and the maximum serum lactate value in CKD subgroup; **(B)** ROC of the maximum phosphate value, the maximum clinical score, and the maximum serum lactate value in non-CKD subgroup; **(C)** ROC of the maximum phosphate value combined with the maximum clinical score and the maximum serum lactate value in CKD subgroup; **(D)** ROC of the maximum phosphate value combined with the maximum clinical score and the maximum serum lactate value in non-CKD subgroup. Model1= SOFA + phosphate. Model2= SAPSII + phosphate. Model3= LODS + phosphate. Model4= lactate + phosphate. CKD, chronic kidney diseases; P_max, maximum phosphate value; SOFA, sequential organ failure assessment; LODS, logistic organ dysfunction score; SAPSII, simplified acute physiology score II.

The value of the maximum serum phosphate value compared to the maximum clinical score and the maximum blood lactate value for the diagnosis of in-hospital mortality was analyzed using ROC (**Figures 9A, B**). We integrated the maximum phosphate value with the maximum clinical score and the maximum serum lactate value by logistic regression, and made ROC analysis again (**Figures 9C, D**).

## Discussion

There are two main conclusions from this study. On the one hand, in septic patients without CKD, the risk of death and shock is significantly higher in patients with hypophosphatemia than in patients with normophosphatemia. Hypophosphatemia is an independent risk factor for death. The risk of death is higher when serum phosphate is lower. On the other hand, among all septic patients, those with hyperphosphatemia have significantly higher severity of disease than those with normophosphatemia. Hyperphosphatemia is an independent risk factor for death. When serum phosphate is higher, the risk of death is greater.

The causes of the occurrence of hypophosphatemia or hyperphosphatemia are classified as acute and chronic. Septic patients are essentially acute in onset. Acute hypophosphatemia is more prevalent in severely ill patients, where it accounts for between 30% and 50% of cases (16). It is commonly seen in the following clinical situations: acute respiratory alkalosis (17), sepsis, alcohol withdrawal or ingestion, and refeeding of malnourished patients (18, 19). Hypophosphatemia accounted for approximately 18% of septic patients in our study. This is not a low proportion. Several studies have shown an association between hypophosphatemia and increased mortality (20–23). It has also been suggested that hypophosphatemia only reflects the severity of the disease and does not increase mortality (4, 24). Given the incidence of hypophosphatemia in sepsis, there is a need to investigate the relationship between hypophosphatemia and the prognosis of sepsis in depth.

Two recent studies have both concluded that hyperphosphatemia is an independent risk factor for death, not hypophosphatemia. Wang H et al. (11) studied the initial values of serum phosphate in 4767 septic patients after admission to the ICU, which showed that hyperphosphatemia (>4.5 mg/dL) was significantly associated with in-hospital mortality (OR 1.48, 95% CI: 1.19-1.85, p=0.001), the association between hypophosphatemia (< 2.5 mg/dL) and in-hospital mortality was not significant (OR 0.91, 95%CI: 0.70-1.19, p=0.145). Shmeylan A et al. (12) studied the initial value of serum phosphate in 1422 septic patients after admission to ICU. The results showed that hyperphosphatemia (> 4.5mg/dL) was significantly associated with hospital mortality (OR 1.7, 95%CI: 1.21-2.29, p = 0.002). There was no significant correlation between hypophosphatemia (< 2.5mg/dL) and hospital mortality (OR 0.89, 95%CI: 0.57-1.39, p =0.59).

Differences in study results may be related to the size of the sample and differences in correction indicators in the multifactorial analysis. 811 (17%) cases in the study by Wang H et al. were classified as hypophosphatemia. 188 (13%) cases in the study by Shmeylan A et al. were classified as hypophosphatemia. 4188 (18%) cases in our study were classified as hypophosphatemia. In addition to demographic characteristics, comorbidities and SOFA scores, Wang H et al. added laboratory data, vasopressin use, and mechanical ventilation as correction factors. The study by Shmeylan A et al. corrected for age, APACHE, gender, and creatinine as variables. Our study performed a subgroup analysis based on whether the patient had combined CKD or not, with the types of initial ICU admission added to the correction criteria. We believe that the types of initial ICU admission to some extent reflects differences in the patients’ primary disease and subsequent treatment. We did not include vasopressin use and mechanical ventilation. Because we thought that the level of serum phosphate might contribute to both and might have some statistical collinearity. In addition, we also did PSM analysis and cox regression to further validate our conclusions. Of course, the above analyses do not fully explain the differences in the study results.

There were also studies that were similar to our results, which suggested that hypophosphatemia was a risk factor for death from sepsis or infectious diseases. Shor R et al. (13) conducted a small study of 55 septic patients, including 26 cases with severe hypophosphatemia (< 1mg/dL) as well as 29 cases with non-severe hypophosphatemia (1-2.5 mg/dL). The results showed that 80.8% of patients with severe hypophosphatemia died and 34.5% of patients with non-severe hypophosphatemia died. The risk of death from severe hypophosphatemia was increased nearly 8-fold (OR 7.89; 95% CI: 2.3-27.6,p=0.001). Mohammad E et al. (14) analyzed 3894 patients with community-acquired pneumonia. The results showed that compared to the normophosphatemia group, the severe hypophosphatemia group (<1.5 mg/dL) and hyperphosphatemia group (>4.5 mg/dL) were significantly associated with a 30-day mortality rate of 38% (OR 2.9, 95%CI: 1.8-4.9, P=0.001) and 39% (OR 3.4, 95%CI: 2.7-4.2, P=0.001).

There are three main mechanisms of hypophosphatemia: decreased absorption of phosphate by the intestine, transfer of phosphate from extracellular to intracellular, increased excretion of phosphate by the kidney, or a combination of these mechanisms (25). The mechanism of hypophosphatemia in the early stages of sepsis should be based on the extracellular transfer of phosphate. Respiratory alkalosis during sepsis increases intracellular pH and subsequently stimulates glycolysis, which leads to accelerated production of phosphorylated metabolites and rapid entry of phosphate into the cell (17, 26). Some studies have also shown that there is also a significant negative correlation between inflammatory cytokines and serum phosphate values. In patients with positive blood cultures, elevated inflammatory factors may cause a decrease in serum phosphate levels (27).

In our study, in the non-CKD subgroup, the risk of death and the occurrence of shock were significantly higher in the hypophosphatemia patients than in the normophosphatemia group, both before and after PSM. Other indicators of the degree of response to the disease were not significantly different (**Tables 1**, **2**). This may be due to the fact that hypophosphatemia leads to inadequate ATP synthesis and subsequently affects the prognosis of sepsis. Insufficient ATP synthesis mainly leads to the development of shock and death. Therefore, the differences in other prognostic indicators were not significant. To further assess the relationship between hypophosphatemia and mortality, we performed a multifactorial logistic regression analysis based on in-hospital mortality and a Cox regression analysis based on 28-day mortality. Both results confirmed that hypophosphatemia was a risk factor for mortality in the non-CKD subgroup, the lower the serum phosphate. The risk of death was greater when serum phosphate was lower. In the CKD subgroup, non-severe hypophosphatemia (1.5-2.7 mg/dL) remained an independent risk factor for death. However, in severe hypophosphatemia (<1.5 mg/dL), this difference was not significant. This could account for the inadequate sample size. Even with our large sample size, only 33 cases of severe hypophosphatemia occurred in patients with combined CKD.

There might also be a number of other reasons why hypophosphatemia affects the prognosis of critically ill patients. Some studies have shown that hypophosphatemia is associated with respiratory muscle dysfunction, which may lead to acute respiratory failure, or prolonged mechanical ventilation (28, 29). However, the occurrence of mechanical ventilation was only different in the CKD subgroup in our study. It has been shown that hypophosphatemia is a significant predictor of ventricular tachycardia (30), which correlates with arrhythmias in septic patients (31). It has been shown that severe hypophosphatemia leads to a 50% reduction in chemotactic phagocytic and bactericidal activity of granulocytes. This resulted in lower granulocyte ATP levels and reduced neutrophil survival (32). These might all contribute to the prognostic impact of hypophosphatemia on sepsis.

In contrast to hypophosphatemia, hyperphosphatemia has rarely been rigorously studied in the sepsis or critically ill patient population (15). However, the available studies all agree that hyperphosphatemia is an independent factor affecting mortality. It has been generally accepted that acute hyperphosphatemia usually occurs in specific settings, such as acute kidney injury, rhabdomyolysis, hemolysis and transcytosis in tumor lysis syndrome (33). Our study showed a total of 3249 (14%) cases septic patients developed hyperphosphatemia, including 1091 (27%) cases in CKD group and 2158 (11%) in non-CKD group. This is not a low percentage. So in this study, we not only graded the degree of hyperphosphatemia, but also extracted the initial and maximum values of serum phosphate respectively after admission to hospital. The aim is to fully investigate the relationship between hyperphosphatemia and the prognosis of sepsis.

There are generally two mechanisms of hyperphosphatemia, endogenous or exogenous. Exogenous refers mainly to increased extracellular phosphate load or reduced renal phosphate excretion. Endogenous mainly refers to trans-cellular transfer of phosphate from the intracellular compartment to the extracellular compartment, which also includes excessive bone resorption or defective bone mineralization (34). There are 2 possible causes of hyperphosphatemia due to sepsis. It is mainly due to the pyroptosis or cell necrotic apoptosis caused by early inflammatory reaction, which leads to cell destruction, and phosphate is transferred from intracellular to extracellular. The second is due to acute kidney injury. Elevated serum phosphate can in turn further promote apoptosis and inflammatory cytokines releasing oxidative stress and exacerbating sepsis (35).

The severity of disease was significantly higher in the hyperphosphatemia group than in the normophosphatemia patients in 2 subgroups, both before and after PSM (**Tables 1**, **3**). This may be because hyperphosphatemia reflects, to some extent, the degree of cellular destruction caused by inflammation. The more heavily infected the cells are, the greater the destruction and the greater the phosphate transfer will be. Therefore, the degree of hyperphosphatemia is directly related to the initial severity of sepsis. As can be broadly seen from the lowess curves and bar graphs, mortality is significantly increased with increasing serum phosphate after greater than normal values. This risk is more pronounced in the maximum values of serum phosphate (**Figure 3**). We graded the initial values of serum phosphate. We also used the normophosphatemia group as the reference group and the other groups as dummy variables. Firstly, a logistic regression analysis was performed based on in-hospital mortality, which showed that the higher the serum phosphate, the greater the risk of death (**Figures 4**, **5**). MIMIC-IV database does not currently have access to 90-day survival information, so we chose the survival rate of 28-days for survival analysis. The K-M curve provides an initial response to the relationship between serum phosphate values and survival. It is clear that the survival rate decreases with increasing serum phosphate values, both for the initial and maximum values of serum phosphate, and even more so for the maximum value of serum phosphate (**Figure 6**). This trend was still evident after the Cox regression analysis (**Figures 7**, **8**). K-M curves were already reflective of the single factor situation, so we did not do another single factor analysis. For the multifactorial analysis, we chose to correct for this with the stepwise backward method, whether it was logistic regression or Cox regression, and the results were consistent. This study focuses on the relationship between the initial value of serum phosphate and mortality. Therefore, we only looked at the relationship between the maximum value of serum phosphate and mortality in general using the lowess curve and the K-M curve, and did not do further regression analysis. The trend is already very clear, i.e. when serum phosphate is higher, mortality is higher. It is clear from our results that hyperphosphatemia is closely related to sepsis. It not only responds to severity, but is also an independent risk factor for mortality. This is related to the mechanism by which hyperphosphatemia arises in sepsis as we mentioned above.

We analyzed the influence of dynamic changes of serum phosphate on prognosis during hospitalization. Compared with the normophosphatemia during hospitalization, the change of serum phosphate will affect the prognosis, and the results of logistic regression and cox regression are consistent. Regardless of the initial value of serum phosphate, patients with hyperphosphatemia after treatment have higher OR and HR values. (**Table 4**).

Because the lowess curve showed a linear relationship between maximum serum phosphate value and mortality, we used the ROC to analyse the diagnostic value of maximum serum phosphate value for in-hospital mortality. And we selected the maximum value of several common critical illness scores and the maximum value of lactate for comparison. In the MIMIC-IV database, LODS score was measured only once, so it was no maximum value. Although the AUC of the maximum serum phosphate value was approximately equal to 0.69, which was of average diagnostic value. It was not inferior to the common predictors such as critical illness scores or lactate values, which was less costly and easier to measure than other indicators. We integrated the maximum phosphate value with the maximum clinical score and the maximum serum lactate value by logistic regression, and made ROC analysis again, it can be seen that AUC has increased after re-integration, but it was still not very ideal (**Figures 9C, D**). The initial serum phosphate values were not linearly related to mortality, so ROC curves were not plotted.

The strengths of our study were the large sample size, the subgroup analysis, the grading of serum phosphate values, the analysis in terms of two aspects of hypophosphatemia and hyperphosphatemia, as well as the correction for possible confounding factors. But it also had limitations. Firstly, because there were only 33 cases of severe hypophosphatemia in the CKD group. The results were likely to be biased. We were unable to determine the relationship between severe hypophosphatemia and mortality in patients with CKD. Secondly, factors influencing the prognosis of sepsis should also include differences in primary disease and differences in treatment, indicators that are difficult to extract in MIMIC-IV, which we can only correct them according to the admission types of different ICUs. Thirdly, this study was retrospective and the causal relationship between serum phosphate and mortality could not be determined.

## Conclusions

In septic patients without chronic kidney disease, the risk of death and shock is higher in patients with hypophosphatemia than in patients with normophosphatemia. Hypophosphatemia is an independent risk factor for death. The risk of death is higher when serum phosphate is lower. In all septic patients, the severity of disease is significantly higher in patients with hyperphosphatemia than in patients with normophosphatemia. Hyperphosphatemia is an independent risk factor for death. When serum phosphate is higher, the risk of death is greater. Both hypophosphatemia and hyperphosphatemia are present in some proportion in sepsis. Clinicians should be aware of the importance of serum phosphate disorders in sepsis. Further studies could investigate whether early correction of serum phosphate disorders could improve the prognosis of sepsis.

## Data availability statement

The original contributions presented in the study are included in the article/**Supplementary Material**. Further inquiries can be directed to the corresponding author.

## Author contributions

WC and ZL conceived the research. TL and ZL extracted data. ZL analyzed the data and wrote the manuscript. CL and YD verified the results. WC revised the manuscript. All authors contributed to the article and approved the submitted version.

## Conflict of interest

The authors declare that the research was conducted in the absence of any commercial or financial relationships that could be construed as a potential conflict of interest.

## Publisher’s note

All claims expressed in this article are solely those of the authors and do not necessarily represent those of their affiliated organizations, or those of the publisher, the editors and the reviewers. Any product that may be evaluated in this article, or claim that may be made by its manufacturer, is not guaranteed or endorsed by the publisher.

## References

[1] Rhodes A Evans LE Alhazzani W Levy MM Antonelli M Ferrer R. Surviving sepsis campaign: International guidelines for management of sepsis and septic shock: 2016. Crit Care Med (2017) 45(3):486–552. doi: 10.1097/CCM.0000000000002255 28098591

[2] Grande E Grippo F Frova L Pantosti A Pezzotti P Fedeli U. The increase of sepsis-related mortality in Italy: A nationwide study, 2003-2015. Eur J Clin Microbiol Infect Dis (2019) 38(9):1701–8. doi: 10.1007/s10096-019-03601-3 31187308

[3] Cawcutt KA Peters SG. Severe sepsis and septic shock: Clinical overview and update on management. Mayo Clin Proc (2014) 89(11):1572–8. doi: 10.1016/j.mayocp.2014.07.009 25444488

[4] Gaasbeek A Meinders AE. Hypophosphatemia: An update on its etiology and treatment. Am J Med (2005) 118(10):1094–101. doi: 10.1016/j.amjmed.2005.02.014 16194637

[5] Leaf DE Wolf M. A physiologic-based approach to the evaluation of a patient with hyperphosphatemia. Am J Kidney Dis (2013) 61(2):330–6. doi: 10.1053/j.ajkd.2012.06.026 PMC5505500 22938849

[6] Prié D Friedlander G. Genetic disorders of renal phosphate transport. N Engl J Med (2010) 362(25):2399–409. doi: 10.1056/NEJMra0904186 20573928

[7] Thompson Bastin ML Adams PM Nerusu S Morris PE Mayer KP Neyra JA. Association of phosphate containing solutions with incident hypophosphatemia in critically ill patients requiring continuous renal replacement therapy. Blood Purif (2022) 51(2):122–9. doi: 10.1159/000514418 33915554

[8] Reintam Blaser A Gunst J Ichai C Casaer MP Benstoem C Besch G. Hypophosphatemia in critically ill adults and children - a systematic review. Clin Nutr (2021) 40(4):1744–54. doi: 10.1016/j.clnu.2020.09.045 33268142

[9] von Landenberg P Shoenfeld Y. New approaches in the diagnosis of sepsis. Isr Med Assoc J (2001) 3(6):439–42. 11433639

[10] Riedler GF Scheitlin WA. Hypophosphataemia in septicaemia: Higher incidence in gram-negative than in gram-positive infections. Br Med J (1969) 1(5646):753–6. doi: 10.1136/bmj.1.5646.753 PMC1982839 4890205

[11] Wang H Zhang L Liao W Huang J Xu J Yang J. Hyperphosphatemia rather than hypophosphatemia indicates a poor prognosis in patients with sepsis. Clin Biochem (2021) 91:9–15. doi: 10.1016/j.clinbiochem.2021.01.016 33600802

[12] Al Harbi SA Al-Dorzi HM Al Meshari AM Tamim H Abdukahil SAI Sadat M. Association between phosphate disturbances and mortality among critically ill patients with sepsis or septic shock. BMC Pharmacol Toxicol (2021) 22(1):30. doi: 10.1186/s40360-021-00487-w 34049590 PMC8161900

[13] Shor R Halabe A Rishver S Tilis Y Matas Z Fux A. Severe hypophosphatemia in sepsis as a mortality predictor. Ann Clin Lab Sci (2006) 36(1):67–72. 16501239

[14] Naffaa ME Mustafa M Azzam M Nasser R Andria N Azzam ZS. Serum inorganic phosphorus levels predict 30-day mortality in patients with community acquired pneumonia. BMC Infect Dis (2015) 15:332. doi: 10.1186/s12879-015-1094-6 26268323 PMC4535260

[15] Miller CJ Doepker BA Springer AN Exline MC Phillips G Murphy CV. Impact of serum phosphate in mechanically ventilated patients with severe sepsis and septic shock. J Intensive Care Med (2020) 35(5):485–93. doi: 10.1177/0885066618762753 29519205

[16] Broman M Wilsson AMJ Hansson F Klarin B. Analysis of hypo- and hyperphosphatemia in an intensive care unit cohort. Anesth Analg (2017) 124(6):1897–905. doi: 10.1213/ane.0000000000002077 28525508

[17] Brautbar N Leibovici H Massry SG. On the mechanism of hypophosphatemia during acute hyperventilation: Evidence for increased muscle glycolysis. Miner Electrolyte Metab (1983) 9(1):45–50. doi: 10.1016/0026-0495(83)90065-3 6843518

[18] Knochel JP Caskey JH. The mechanism of hypophosphatemia in acute heat stroke. JAMA (1977) 238(5):425–6. doi: 10.1001/jama.1977.03280050065027 577561

[19] Marik PE Bedigian MK. Refeeding hypophosphatemia in critically ill patients in an intensive care unit. A Prospective Study. Arch Surg (1996) 131(10):1043–7. doi: 10.1001/archsurg.1996.01430220037007 8857900

[20] Chung PY Sitrin MD Te HS. Serum phosphorus levels predict clinical outcome in fulminant hepatic failure. Liver Transpl (2003) 9(3):248–53. doi: 10.1053/jlts.2003.50053 12619021

[21] Cohen J Kogan A Sahar G Lev S Vidne B Singer P. Hypophosphatemia following open heart surgery: Incidence and consequences. Eur J Cardiothorac Surg (2004) 26(2):306–10. doi: 10.1016/j.ejcts.2004.03.004 15296888

[22] Hoffmann M Zemlin AE Meyer WP Erasmus RT. Hypophosphataemia at a Large academic hospital in south Africa. J Clin Pathol (2008) 61(10):1104–7. doi: 10.1136/jcp.2007.054940 18820097

[23] Zazzo JF Troché G Ruel P Maintenant J. High incidence of hypophosphatemia in surgical intensive care patients: Efficacy of phosphorus therapy on myocardial function. Intensive Care Med (1995) 21(10):826–31. doi: 10.1007/bf01700966 8557871

[24] Geerse DA Bindels AJ Kuiper MA Roos AN Spronk PE Schultz MJ. Treatment of hypophosphatemia in the intensive care unit: A review. Crit Care (2010) 14(4):R147. doi: 10.1186/cc9215 20682049 PMC2945130

[25] Amanzadeh J Reilly RF Jr. Hypophosphatemia: An evidence-based approach to its clinical consequences and management. Nat Clin Pract Nephrol (2006) 2(3):136–48. doi: 10.1038/ncpneph0124 16932412

[26] Relman AS. Metabolic consequences of acid-base disorders. Kidney Int (1972) 1(5):347–59. doi: 10.1038/ki.1972.46 4275852

[27] Barak V Schwartz A Kalickman I Nisman B Gurman G Shoenfeld Y. Prevalence of hypophosphatemia in sepsis and infection: The role of cytokines. Am J Med (1998) 104(1):40–7. doi: 10.1016/s0002-9343(97)00275-1 9528718

[28] Gravelyn TR Brophy N Siegert C Peters-Golden M. Hypophosphatemia-associated respiratory muscle weakness in a general inpatient population. Am J Med (1988) 84(5):870–6. doi: 10.1016/0002-9343(88)90065-4 3364446

[29] Aubier M Murciano D Lecocguic Y Viires N Jacquens Y Squara P. Effect of hypophosphatemia on diaphragmatic contractility in patients with acute respiratory failure. N Engl J Med (1985) 313(7):420–4. doi: 10.1056/nejm198508153130705 3860734

[30] Ognibene A Ciniglio R Greifenstein A Jarjoura D Cugino A Blend D. Ventricular tachycardia in acute myocardial infarction: The role of hypophosphatemia. South Med J (1994) 87(1):65–9. doi: 10.1097/00007611-199401000-00014 7506845

[31] Schwartz A Gurman G Cohen G Gilutz H Brill S Schily M. Association between hypophosphatemia and cardiac arrhythmias in the early stages of sepsis. Eur J Intern Med (2002) 13(7):434. doi: 10.1016/s0953-6205(02)00130-9 12384132

[32] Craddock PR Yawata Y VanSanten L Gilberstadt S Silvis S Jacob HS. Acquired phagocyte dysfunction. a complication of the hypophosphatemia of parenteral hyperalimentation. N Engl J Med (1974) 290(25):1403–7. doi: 10.1056/nejm197406202902504 4208370

[33] Qadeer HA Bashir K. Physiology, phosphate. In: Statpearls. Treasure Island (FL: StatPearls Publishing Copyright© 2022, StatPearls Publishing LLC (2022).

[34] Koumakis E Cormier C Roux C Briot K. The causes of hypo- and hyperphosphatemia in humans. Calcif Tissue Int (2021) 108(1):41–73. doi: 10.1007/s00223-020-00664-9 32285168

[35] Voelkl J Lang F Eckardt KU Amann K Kuro OM Pasch A. Signaling pathways involved in vascular smooth muscle cell calcification during hyperphosphatemia. Cell Mol Life Sci (2019) 76(11):2077–91. doi: 10.1007/s00018-019-03054-z PMC6502780 30887097

